# Continuous Exposure to Low-Dose-Rate Gamma Irradiation Reduces Airway Inflammation in Ovalbumin-Induced Asthma

**DOI:** 10.1371/journal.pone.0143403

**Published:** 2015-11-20

**Authors:** Joong Sun Kim, Yeonghoon Son, Min Ji Bae, Seung Sook Lee, Sun Hoo Park, Hae June Lee, Soong In Lee, Chang Geun Lee, Sung Dae Kim, Wol Soon Jo, Sung Ho Kim, In Sik Shin

**Affiliations:** 1 Research Center, Dongnam institute of Radiological & Medical Sciences (DIRAMS), Busan, South Korea; 2 Laboratory of Radiation Exposure & Therapeutics, Seoul, South Korea; 3 Division of Radiation effect, Korea institute of Radiological & Medical Sciences (KIRAMS), Seoul, South Korea; 4 College of Oriental Medicine, Dongshin University, Jeonnam, South Korea; 5 College of Veterinary Medicine, Chonnam National University, Gwangju, South Korea; French National Centre for Scientific Research, FRANCE

## Abstract

Although safe doses of radiation have been determined, concerns about the harmful effects of low-dose radiation persist. In particular, to date, few studies have investigated the correlation between low-dose radiation and disease development. Asthma is a common chronic inflammatory airway disease that is recognized as a major public health problem. In this study, we evaluated the effects of low-dose-rate chronic irradiation on allergic asthma in a murine model. Mice were sensitized and airway-challenged with ovalbumin (OVA) and were exposed to continuous low-dose-rate irradiation (0.554 or 1.818 mGy/h) for 24 days after initial sensitization. The effects of chronic radiation on proinflammatory cytokines and the activity of matrix metalloproteinase-9 (MMP-9) were investigated. Exposure to low-dose-rate chronic irradiation significantly decreased the number of inflammatory cells, methylcholine responsiveness (PenH value), and the levels of OVA-specific immunoglobulin E, interleukin (IL)-4, and IL-5. Furthermore, airway inflammation and the mucus production in lung tissue were attenuated and elevated MMP-9 expression and activity induced by OVA challenge were significantly suppressed. These results indicate that low-dose-rate chronic irradiation suppresses allergic asthma induced by OVA challenge and does not exert any adverse effects on asthma development. Our findings can potentially provide toxicological guidance for the safe use of radiation and relieve the general anxiety about exposure to low-dose radiation.

## Introduction

The recent nuclear accident in Japan has increased public concern about the critical effects of radiation exposure. High-dose and high-dose-rate radiation have been shown to induce detrimental effects in various organisms, thereby causing cell death [1]. In contrast, low-dose radiation has been reported to exert various beneficial effects [2, 3]. These distinct effects are proportional to the dose and rate of irradiation [4, 5]. Low-dose radiation exposure has been a rising issue in modern society because many people are potentially exposed to it. The safe dose of this type of radiation has been determined, and the effects of low-dose (≤0.3 Gy) and low-dose-rate (≤6 mGy/h) radiation have been investigated according to the recommendations of the United Nations Scientific Committee on the Effects of Atomic Radiation [6]. Nonetheless, the effects of low-dose radiation exposure on the development of diseases have not been examined. Further investigations on the correlation between low-dose radiation and disease development are therefore warranted in order to relieve the anxiety of the public.

Asthma is a chronic inflammatory airway disease. Its prevalence has increased in recent decades, and it generally occurs during childhood or young adulthood [7]. It is generally caused by the inhalation of allergens such as pollens, house dust, inhalants, and air pollutants and is characterized by eosinophilic airway inflammation, airway hyper-responsiveness, and mucus hypersecretion [8]. Inflammatory cells release various chemical mediators that are closely associated with asthma development [9]. Various studies have demonstrated the relationship between radiation exposure and the development of asthma [10–12], but the results have been contradictory. While one study reported that radiation exposure induced chronic airway inflammation, another study demonstrated that the asthmatic response was attenuated by radiation [13, 14]. These conflicting results have been attributed to the radiation dose and rate used.

To clearly determine the correlation between low-dose-rate radiation and asthma, we investigated the effects of continuous low-dose-rate irradiation on disease development in murine model of ovalbumin (OVA)-induced asthma. In addition, various biological assays, including immunohistochemistry, western blotting, and enzyme-linked immunosorbent assay (ELISA), were conducted to elucidate the mechanism of action of low-dose-rate radiation.

## Materials and Methods

### Animals

Six-week-old female C57BL/6 mice (Central Lab. Animal Inc., Seoul, Korea) were used after one week of quarantine and acclimatization. The animals were maintained in a room at 23 ± 2°C, with a relative humidity of 50 ± 5%, artificial lighting from 08:00–20:00 and 13~18 air changes per hour. The mice were given a standard laboratory diet and water ad libitum. All experimental procedures were carried out in accordance with the NIH Guidelines for the Care and Use of Laboratory Animals and were conducted following a protocol approved by the Institutional Animal Care and Use Committee of the Dongnam Institute of Radiological and Medical Sciences (Permit Number: DI-2015-002). The animals were cared for in accordance with the dictates of the National Animal Welfare Law of Korea.

### Experimental procedure

**Fig 1:**
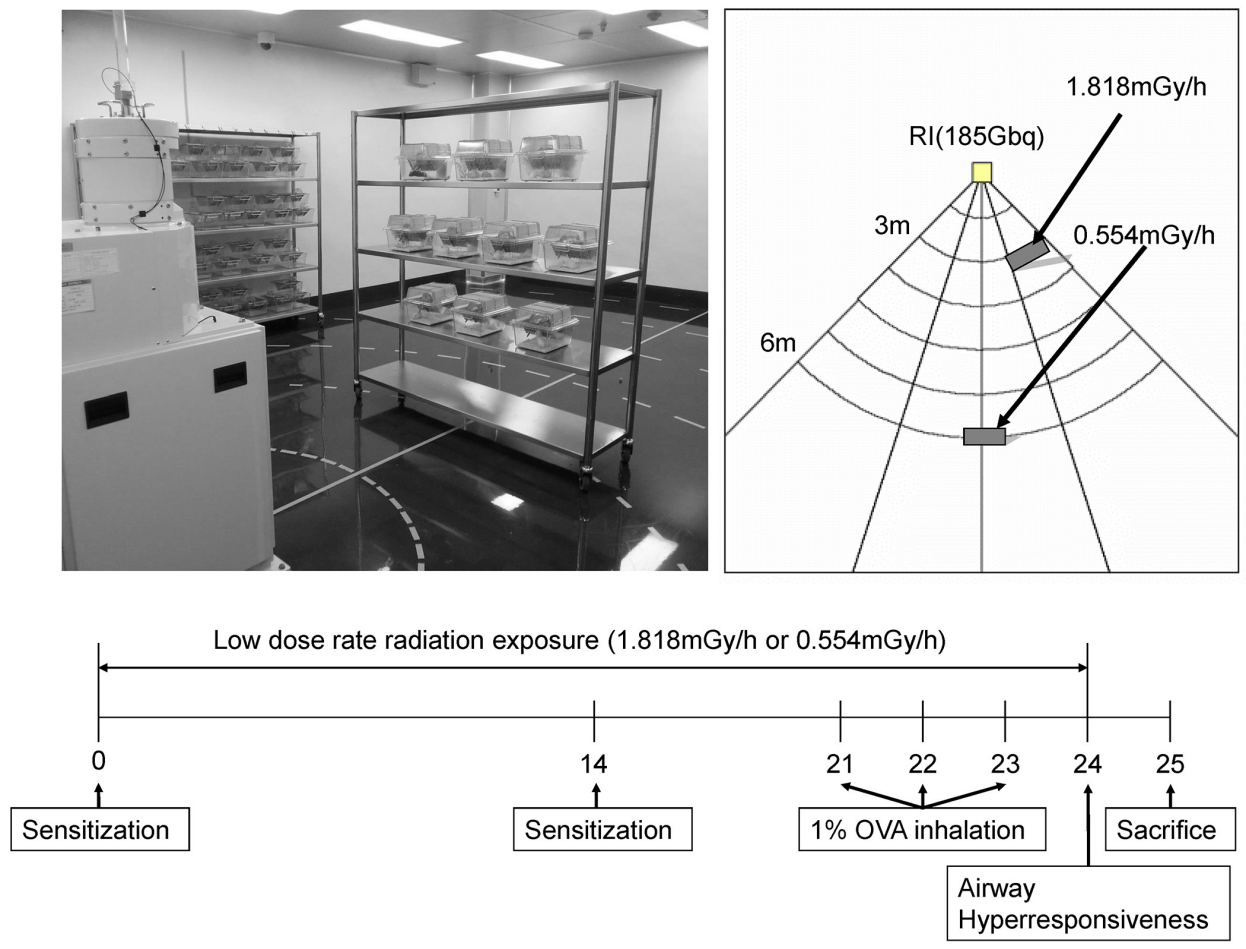
Experimental procedure for establishing the OVA-induced asthma model. (A) Low-dose-rate chronic irradiation equipment at Dongnam Institute of Radiological and Medical Sciences. (B) Schematic diagram of irradiation and experimental procedures.

The mice were randomly divided into six groups (n = 6 per group): sham, 0.3 Gy, 1 Gy, OVA, OVA+0.3 Gy, and OVA+1 Gy. The experimental procedure for the OVA-induced asthma model is described in Fig 1. Briefly, the mice were sensitized on days 0 and 14 with an intraperitoneal injection of 20 μg of OVA (Sigma-Aldrich, Carlsbad, CA, USA) emulsified with 2 mg of aluminum hydroxide in 200 μL of phosphate-buffered saline (PBS) (pH 7.4). On days 21, 22, and 23, the mice received an airway challenge with OVA (1% (w/v)) for 1 h, using an ultrasonic nebulizer (NE-U12; Omron Corp., Tokyo, Japan). Radiation was administered in the long-term low-dose-rate chronic radiation facility at the DIRAMS, and was conducted in a specific pathogen-free conditioned irradiation room equipped with a ^137^Cs source (185 GBq). The mouse cages were placed on shelves located 6 m (0.554 mGy/h) or 3 m (1.818 mGy/h) from the source providing sham, 0.3 Gy (0.554 mGy/h), or 1 Gy (1.818 mGy/h) exposure time for 24 days. The animals were continuously exposed to the radiation for 24 h a day with the exception of 2 h a week during which the room was cleaned, bedding was changed, and the food and water were refreshed. Sham control mice for both treatment groups were placed on shelves in the same facility and shielded from the radiation (Fig 1). The methylcholine responsiveness was assessed 24 h after the final challenge by indirectly using single-chamber, whole body plethysmography (Allmedicus, Seoul, Korea).

### Inflammatory cell count in bronchoalveolar lavage fluid

Bronchoalveolar lavage fluid (BALF) was isolated for analysis. The mice were sacrificed 48 h after the final challenge with an intraperitoneal injection of pentobarbital (50 mg/kg; Hanlim Pharm. Co., Seoul, Korea), and a tracheostomy was performed. In order to obtain the BALF, ice-cold PBS (0.5 mL) was infused into the lungs three times and withdrawn each time through tracheal cannulation (total volume, 1.5 mL). The total inflammatory cell numbers were determined by counting the cells in at least five squares of a hemocytometer after the exclusion of dead cells by Trypan blue staining. The differential cell counts in the BALF were performed using the Diff-Quik® staining reagent (B4132-1A; IMEB, CA, USA), according to the manufacturer’s instructions. The numbers of macrophages, neutrophils, and lymphocytes were calculated by multiplying the percentages obtained by the total yield. Images of slides were taken using a digital camera mounted on a microscope (Nikon Eclipse 80i; Nikon Corporation; Tokyo, Japan).

### Measurement of cytokine levels in BALF and OVA-specific IgE in serum

The levels of IL-4 (sensitivity: 2 pg/mL) and IL-5 (sensitivity: 7 pg/mL) in the BALF were measured using sandwich ELISA kits (R&D System, Minneapolis, MN, USA) according to the manufacturer’s protocols. ELISA was also used to determine the levels of OVA-specific IgE in the serum. Briefly, 96-well microtiter plates were coated overnight with 10 μg/mL OVA in PBS-Tween 20. The plate was washed and blocked, and then the samples were added and the plate was incubated for 2 h. After another washing step, HRP-conjugated goat anti-mouse IgE antibody was added, the plates were washed four times, and 200 μL of o-phenylene diamine dihydrochloride (Sigma-Aldrich) was added to each well. The absorbance was measured at 450 nm after incubation for 10 min in the dark. ELISA was performed three times.

### Histopathology

After isolation of the BALF, the right lung tissue was fixed in 10% buffered formalin and embedded in paraplast wax. Lung tissue sections of 4-μm thickness were prepared for hematoxylin-eosin staining and periodic acid-Schiff (PAS) (IMEB, San Marcos, CA, USA) to estimate the inflammation and mucus production, respectively. Two tissue sections from four different parts of the lung from each animal were prepared for histological examination. Images of lung sections were obtained using a digital camera mounted on a microscope (Nikon Eclipse 80i; Nikon Corporation; Tokyo, Japan). Quantification was performed using Image-Pro Plus image analyzing software (Media Cybernetics; Bethesda, MD, USA). Quantitative index was expressed as the ratio (%) of selected area to the whole histological field.

Each tissue section was evaluated with a score from 0–4 based on the amount of area affected by interstitial inflammation, alveolar wall thickening, peribronchial inflammation, and interstitial edema (0 ≤ 10%, 1 = up to 30%, 2 = up to 50%, 3 = up to 70%, 4 ≥ 70%) [15].

For immunohistochemistry, MMP-9 antigen was assessed with a specific rabbit antibody (4730, 1:100; Abcam, Cambridge, UK). Antigen-antibody complexes were visualized using the avidin-biotin-peroxidase complex kit (Elite kit, Vector, Burlingame, CA, USA). The sections were counterstained with hematoxylin and mounted. Images of intestinal sections were taken with a digital camera mounted on a Nikon Eclipse 80i microscope (Nikon Corporation, Kanagawa, Japan).

### Western blot analysis

The left lung tissue sonicates were solubilized in SDS-polyacrylamide gel electrophoresis sample buffer, and the protein concentration in each sample was determined using a Bio-Rad protein assay kit (Bio-Rad Laboratories, Hercules, CA USA) with bovine serum albumin as the standard. Total protein equivalents for each sample (70 μg protein per lane) were then separated in 10% SDS-polyacrylamide gels and electrophoretically transferred to an Immobilon-P^SQ^ transfer membrane (Roche Diagnostics, IN, USA). The membrane was immediately placed into a blocking solution (5% nonfat milk) at room temperature for 1 h. The membrane was incubated with diluted primary antibodies for Mucin-5 (24071, 1:1,000; Abcam), MMP-9 (4730, 1:1,000; Abcam), p-ERK (4730, 1:1,000; Cell Signaling, Danvers, MA, USA), ERK (4695, 1:1,000, Cell Signaling), p-JNK (9255, 1:1,000; Cell Signaling), JNK (9258, 1:1,000; Cell Signaling), and β-actin (1:10,000, Sigma Aldrich) in TBS-T buffer (Tris-HCl-based buffer with 0.2% Tween 20, pH 7.5) at 4°C overnight. After four washes with TBS-T for 10 min each, the membrane was incubated with the secondary polyclonal anti-rabbit antibody or monoclonal anti-mouse antibody (1:10,000, Sigma Aldrich) in TBS-T buffer at room temperature for 1 h. Horseradish-conjugated secondary antibody labeling was detected by enhanced chemiluminescence and exposure to a radiographic film. Pre-stained blue markers were used for molecular weight determination. The bands were quantified using Scion Image Beta 4.0.2 for Windows XP software (Scion, Frederick, ME, USA). Immunoblot was performed three times.

### Statistical analysis

The data are reported as the mean ± SEM. Non-repeated measures two-way analysis of variance (ANOVA) was used to test for main effect of OVA, radiation dose (0, 0.3, and 1 Gy), and interactions. Newman-Keuls *post hoc* test was used to perform multiple comparisons where warranted by a significant omnibus F-statistic. The results of multiple comparisons are provided in the relevant figures below. In all cases, a *p*-value < 0.05 was considered significant.

## Results

### Chronic low-dose-rate irradiation decreases the recruitment of inflammatory cells in the BALF of OVA-sensitized/challenged mice

**Fig 2:**
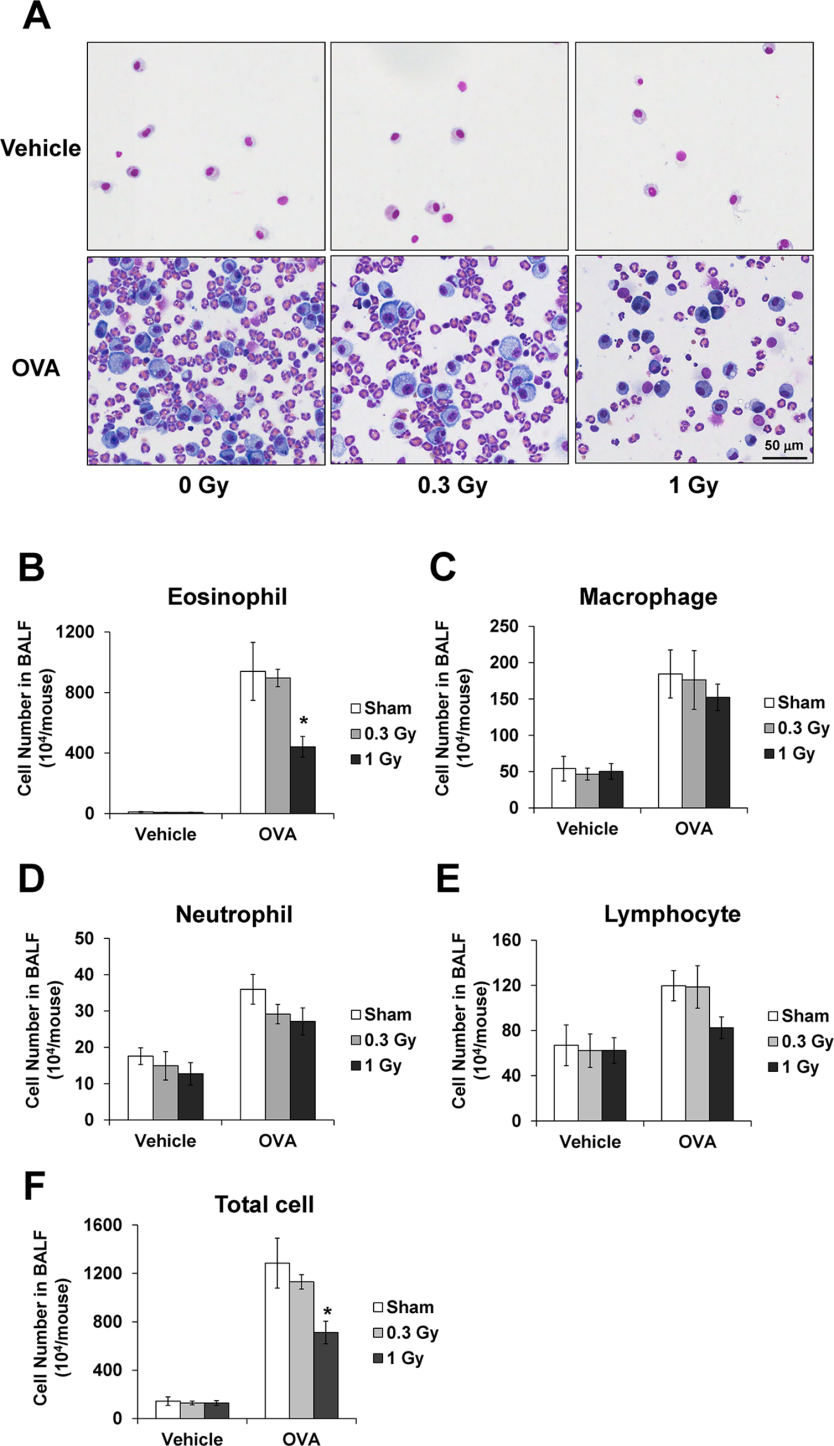
Inflammatory cell count of the bronchoalveolar lavage fluid (BALF) decreases after irradiation in OVA-sensitized/challenged mice. (A) Representative pictures of Diff-quick staining of cytospin preparation. Changes in the number of eosinophils (B), macrophages (C), neutrophils (D), lymphocytes (E), and total cells (F) in the BALF of mice. The data are reported as means ± SE (*n* = 6 per group). *p < 0.05 *vs*. OVA-sensitized/challenged mice.

The number of inflammatory cells including eosinophils, macrophages, lymphocytes, and neutrophils, was significantly increased in the BALF of OVA-sensitized/challenged mice compared with the normal controls (Fig 2). The number of these cells, particularly eosinophils, in the BALF of radiation-exposed mice was markedly decreased compared with the OVA-sensitized/challenged mice.

### Methylcholine responsiveness of OVA-sensitized/challenged mice is reduced by irradiation

**Fig 3:**
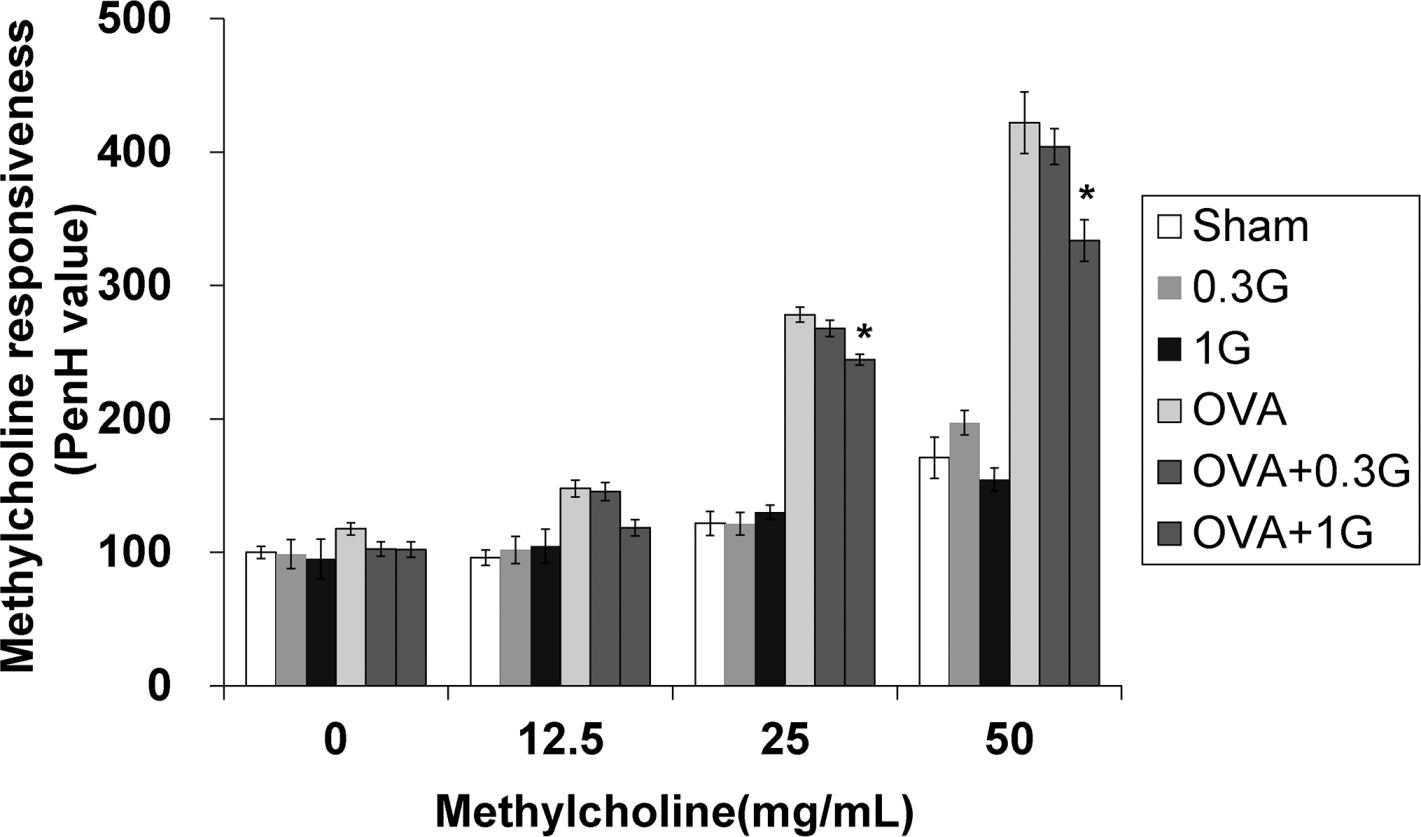
Methylcholine responsiveness (PenH value) is decreased by irradiation in OVA-sensitized/challenged mice. The methylcholine responsiveness was indirectly assessed 24 h after the last challenge using single-chamber, whole body plethysmography (Allmedicus, Seoul, Korea). Continuous exposure to low-dose-rate radiation reduced methylcholine responsiveness compared with that of the OVA-sensitized/challenged mice. The data are reported as means ± SE (*n* = 6 per group). *p < 0.05 *vs*. OVA-sensitized/challenged mice.

The methylcholine responsiveness of the OVA-sensitized/challenged mice was significantly elevated with increasing concentration of methylcholine (12.5–50 mg/mL) compared to that in normal controls (Fig 3). The mice subjected to low-dose-rate chronic irradiation showed significantly lower methylcholine responsiveness than the OVA-challenged mice exposed to methylcholine at doses of 25 and 50 mg/mL.

### Production of proinflammatory cytokines and OVA-specific immunoglobulins is decreased by chronic irradiation

**Fig 4:**
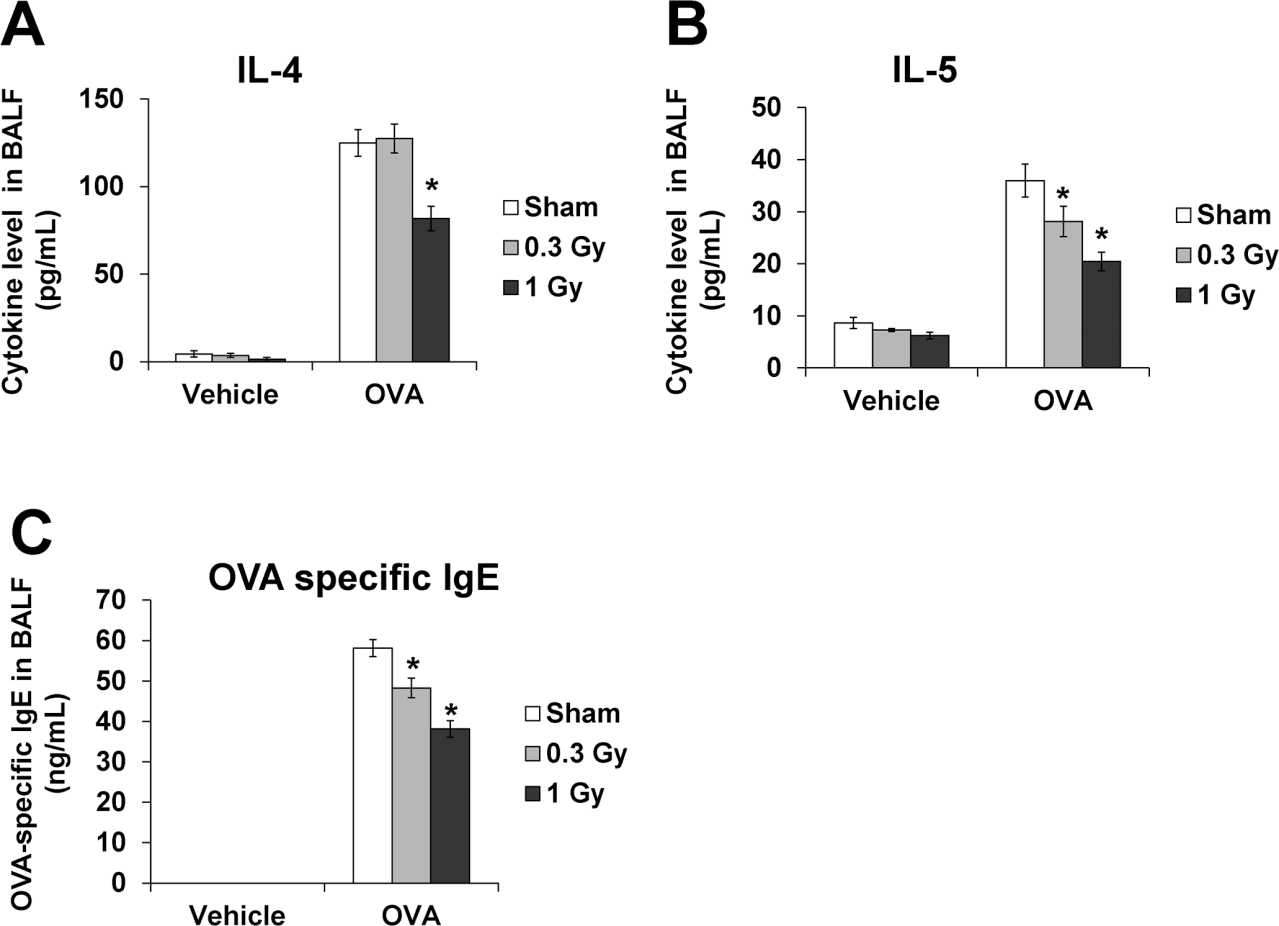
IL-4 and IL-5 levels decreased in the BALF and OVA-specific IgE in the serum from OVA-sensitized/challenged mice after irradiation. The OVA-sensitized/challenged mice showed significant increases in these cytokines. Continuous exposure to low-dose-rate radiation significantly reduced these cytokines compared with that in the OVA-sensitized/challenged mice. The data are reported as means ± SE (*n* = 6 per group). *p < 0.05 *vs*. OVA-sensitized/challenged mice.

The levels of IL-4 were significantly increased in the OVA-sensitized/challenged mice compared with the normal controls (Fig 4A). The mice exposed to low-dose-rate radiation of 1 Gy showed a significant reduction in IL-4 levels compared with the OVA-sensitized/challenged mice. Similar to the results for IL-4, the OVA-sensitized/challenged mice showed markedly increased levels of IL-5 compared with the normal controls, and these levels decreased in the radiation-exposed mice in a dose-dependent manner compared with the OVA-sensitized/challenged mice (Fig 4B).

The level of OVA-specific IgE in the serum was significantly increased in the OVA-sensitized/challenged mice compared with the normal controls (Fig 4C). The mice treated with 1 Gy of low-dose-rate chronic irradiation showed decreased OVA-specific IgE in the serum compared with the OVA-sensitized/challenged mice.

### Low-dose-rate chronic irradiation attenuates airway inflammation induced by OVA-sensitization/challenge

**Fig 5:**
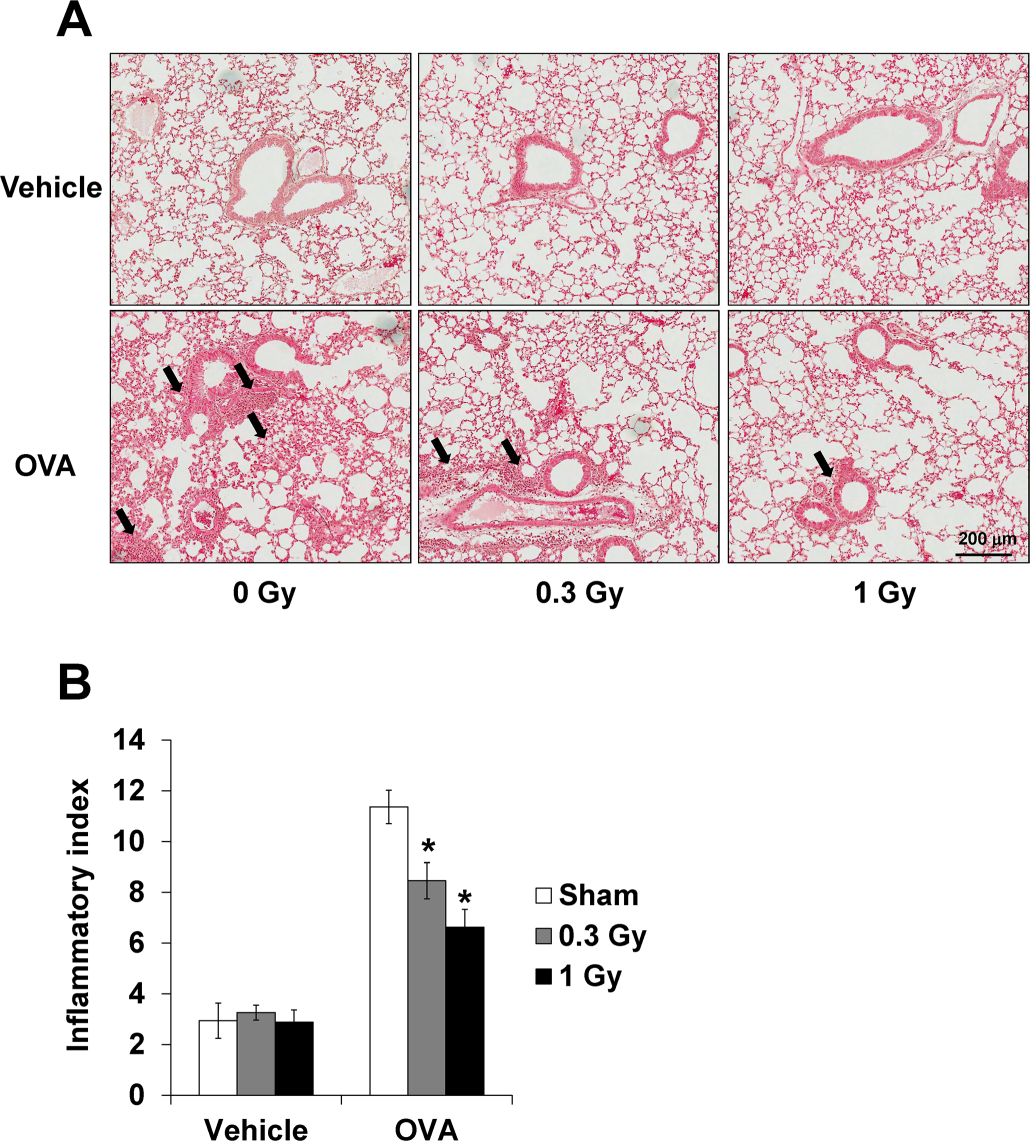
Airway inflammation was attenuated by irradiation in the lung tissue from OVA-sensitized/challenged mice. (A) Histological examination of the airway inflammation (black arrow) stained with H&E (magnification ×200), (B) quantitative analysis for airway inflammation. Continuous exposure to low-dose-rate radiation reduced the airway inflammation in the lung tissue. The data are reported as means ± SE (*n* = 6 per group). *p < 0.05 *vs*. OVA-sensitized/challenged mice.

The OVA-challenged mice showed marked infiltration of inflammatory cells into the peribronchial and perivascular lesions in the lung tissue (Fig 5). After low-dose-rate irradiation, this cell infiltration decreased dose-dependently in radiation-exposed mice relative to that in the OVA-challenged mice.

### Mucus hyper-secretion induced by OVA-sensitization/challenge is attenuated by chronic low-dose-rate irradiation

**Fig 6:**
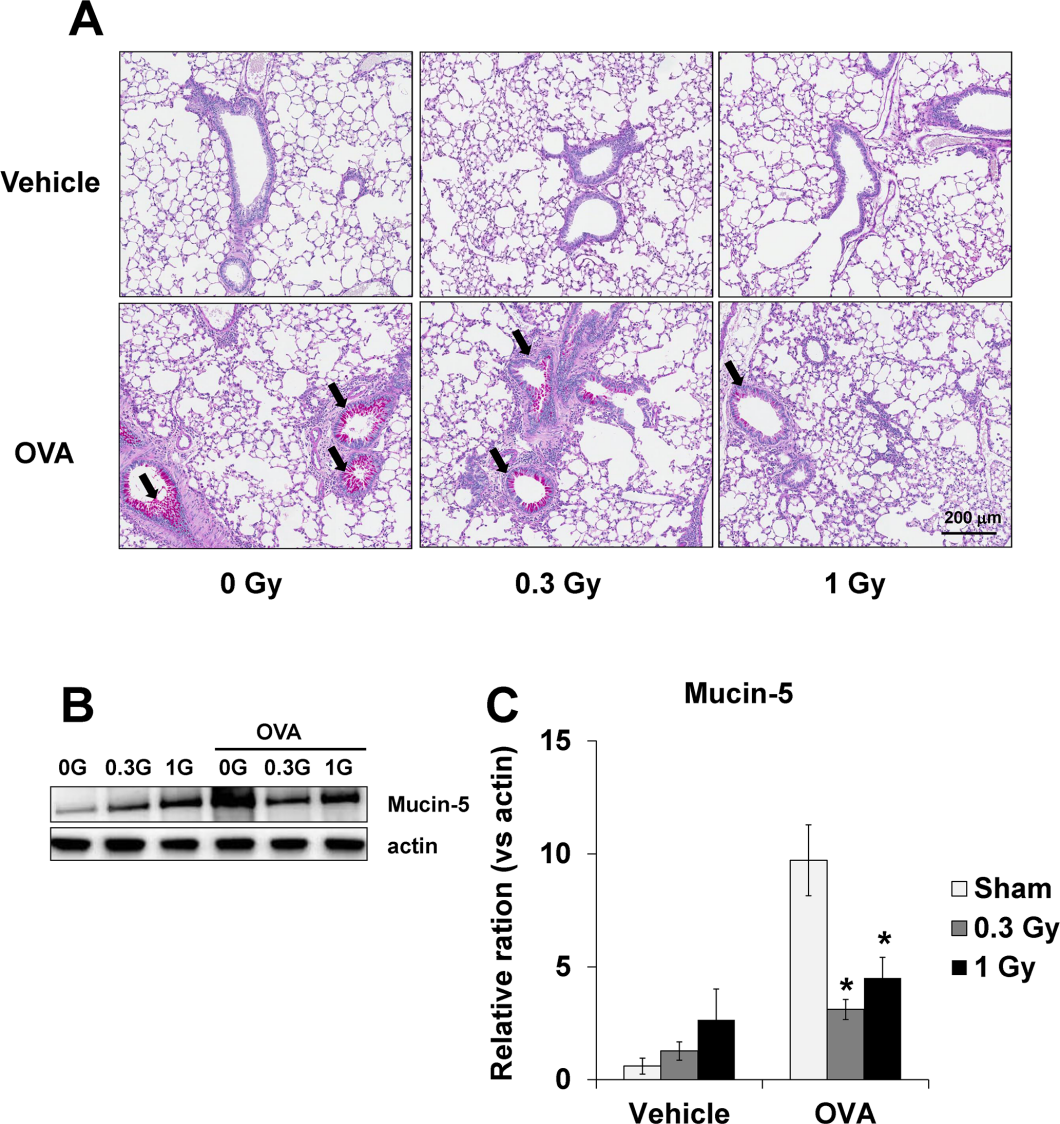
Mucus production decreased following irradiation in the lung tissue from OVA-sensitized/challenged mice. (A) Histological examination of mucus production (black arrow) in the lung tissue stained with PAS (magnification ×200), (B) western blot for Mucin-5, and (C) relative ratio vs. β-actin. Continuous exposure to low-dose-rate radiation showed marked reduction of mucus production and expression of Mucin-5 in the lung tissue. The data are reported as means ± SE (*n* = 6 per group). *p < 0.05 *vs*. OVA-sensitized/challenged mice.

In the lung sections stained with PAS, mucus overproduction was observed in the bronchial airways of the OVA-challenged mice. The mucus production was lower in the lung tissue of mice exposed to low-dose-rate radiation than in the OVA-challenged mice (Fig 6A). These findings were similar to the western blotting results of Mucin-5 expression in lung tissue, where Mucin-5 expression was increased in the OVA-challenged mice, while it was significantly reduced in the radiation-exposed mice (Fig 6B and 6C).

### Low-dose-rate irradiation suppresses MMP-9 expression in the lung tissue of OVA-sensitized/challenged mice

**Fig 7:**
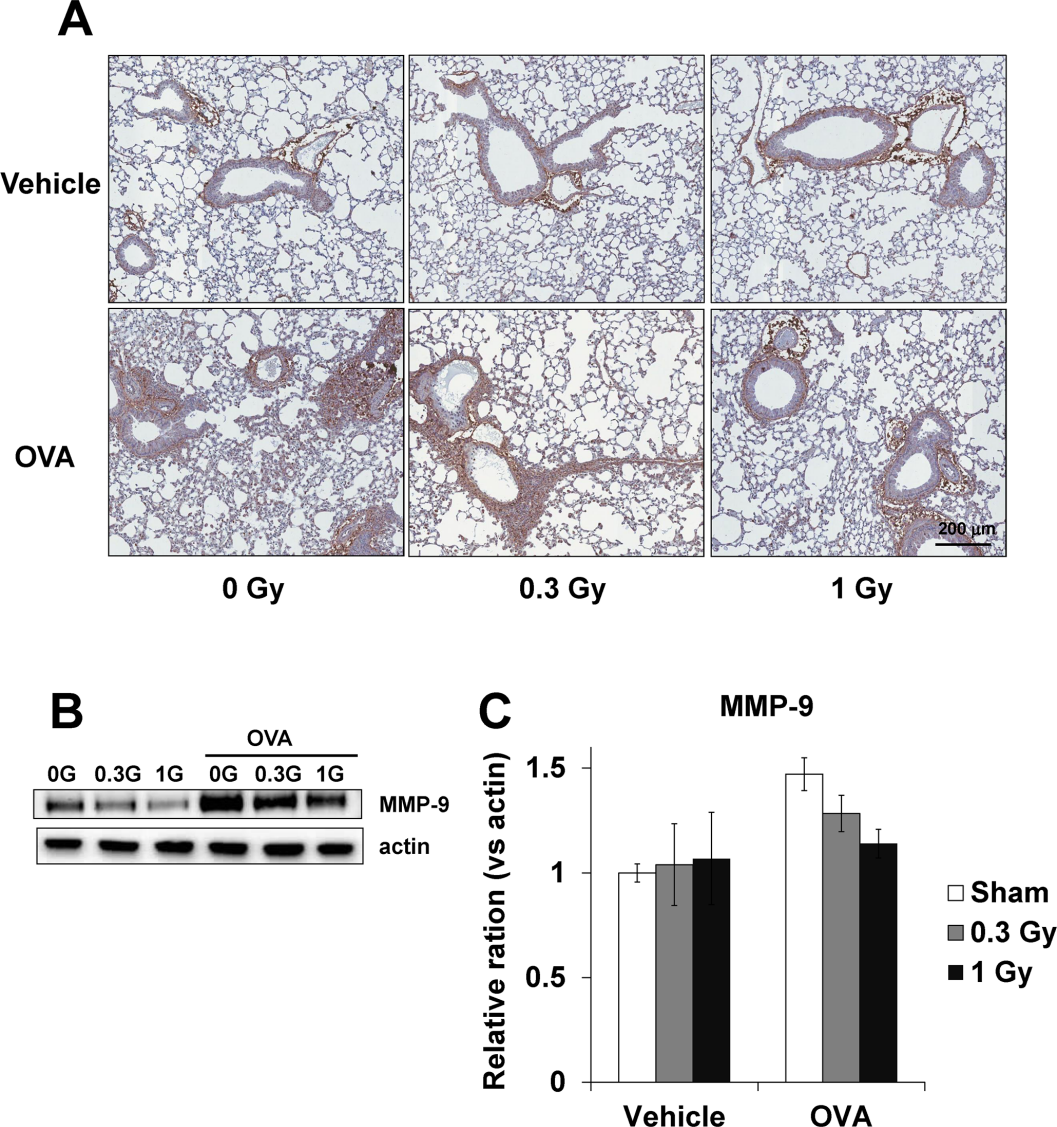
MMP-9 expression in the lung tissue from OVA-sensitized/challenged mice. (A) Immunohistochemical examination of MMP-9 in the lung tissue, (B) western blot for MMP-9, and (C) relative ratio vs. β-actin. The OVA-sensitized/challenged mice exhibited a significant increase in MMP-9 activity and expression in the lung tissue. Continuous exposure to low-dose-rate radiation markedly reduced MMP-9 activity and expression in the lung tissue. The data are reported as means ± SE (*n* = 6 per group). *p < 0.05 *vs*. OVA-sensitized/challenged mice.

The OVA-sensitized/challenged mice showed a significant elevation in MMP-9 expression in the lung tissue compared with the normal controls (Fig 7A). The mice treated with low-dose-rate chronic irradiation mice exhibited a meaningful reduction in MMP-9 expression compared with the OVA-sensitized mice. Similar results were obtained for MMP-9 protein by western blotting (Fig 7B and 7C).

### Phosphorylation of ERK and JNK is suppressed in the lung tissue of OVA-sensitized/challenged mice after irradiation

**Fig 8:**
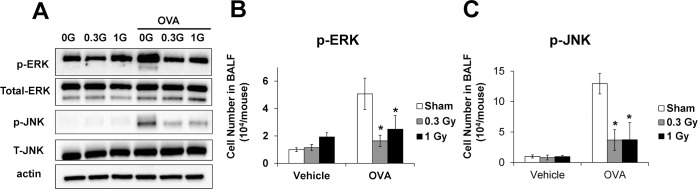
ERK and JNK phosphorylation in the lung tissue from OVA-sensitized/challenged mice. (A) Western blot for ERK and JNK, (B) phosphorylation of ERK (relative ratio vs. β-actin), and (C) phosphorylation of JNK (relative ratio vs. β-actin). The OVA-sensitized/challenged mice exhibited a significant increase in phosphorylation of ERK and JNK in the lung tissue. Continuous exposure to low-dose-rate radiation markedly reduced phosphorylation of ERK and JNK in the lung tissue. The data are reported as means ± SE (*n* = 6 per group). *p < 0.05 *vs*. OVA-sensitized/challenged mice.

ERK and JNK phosphorylation in the lung tissue was increased in the OVA-sensitized/challenged mice compared with normal control. In contrast, the low-dose-rate-irradiated mice showed a significant reduction in ERK and JNK phosphorylation compared with the OVA-sensitized mice (Fig 8).

## Discussion

Ionizing radiation has long been established as harmful to living organisms, but the adverse effects of low doses of radiation remain controversial. The recent nuclear accident in Japan has further increased such anxiety, and exposure to radiation as a potential risk factor for human health has become a growing public concern. Current events throughout the world underscore the potentially harmful effects of environmental, accidental, or therapeutic radiation exposure. A large range of dose rates of ionizing radiations is potentially encountered in accidental radiation exposure. Nevertheless, most of the studies related to radiation effects have only examined a high-dose rate [16]. In this study, the effects of a continuous whole-body exposure to low-dose-rate radiation were evaluated in OVA-challenged C57BL/6 mice.

Low-dose irradiation can ameliorate autoimmune diseases such as arthritis and autoimmune encephalomyelitis [17–19]. However, its effect on asthma remains unclear. In the present study, exposure to low-dose-rate radiation significantly decreased the number of eosinophils in the BALF, methylcholine responsiveness, and the level of OVA-specific IgE in the serum and Th2 cytokines. These findings were accompanied by those of histological analyses of lung tissue, demonstrating a reduction in airway inflammation and mucus production in the lung sections from the mice exposed to low-dose-rate radiation. Based on these results, low-dose-rate irradiation was considered to effectively suppress allergic asthma induced by OVA challenge by downregulating Th2 cytokines.

Allergic asthma is an inflammatory condition of the airway caused by exacerbated responses to inhaled allergens and characterized by reversible airway obstruction, increased mucus production, and infiltration of eosinophils [20]. These alterations are accompanied by changes in biological markers such as cytokines (IL-4 or IL-5) and allergen-specific IgE, which are crucial mediators in the development of asthma. These mediators are associated with recruitment of inflammatory cells, mucus secretion, and methylcholine responsiveness [21]. In the present study, OVA-induced asthmatic mice showed increase in the number of inflammatory cells, cytokine release, OVA-specific IgE levels, and methylcholine responsiveness. By contrast, OVA-induced asthmatic mice treated with low-dose irradiation exhibited reduction in the number of inflammatory cells, including eosinophils, macrophages, and neutrophils, with a concomitant decrease in the levels of IL-4, IL-5, and OVA-specific IgE. In particular, 1 Gy irradiation caused a significant reduction, indicating suppression of asthma development. Histological findings for the lung tissue were also consistent with these results, as OVA-induced asthmatic mice showed extensive infiltration of inflammatory cells into the lung, while mice exposed to low-dose radiation exhibited a significant reduction in the inflammatory response. These results indicate that low-dose irradiation does not aggravate asthmatic responses, but attenuates these effects. A previous study has demonstrated that fractionated irradiation at 5 Gy aggravated the severity of asthma [10], while it was attenuated by 2 Gy irradiation [13, 14]. The difference between these previous reports and the present findings could be due to the differences in the dosage and dose rate of irradiation. The doses used in this study are 0.3 Gy and 1 Gy. Although acute high-dose irradiation causes myelotoxicity, the doses used in this study did not significantly decrease peripheral blood (Supporting information). According to previous literature, 0.3 Gy is a non-toxic dose, and 1 Gy irradiation has been shown to not exert any toxic effects on various organs except the reproductive organs [22, 23]. The available literature on the toxicology of irradiation strongly supports the fact that low-dose irradiation does not promoted asthma development.

MMP-9 is considered an important target in the development of asthmatic disease [24]. The metalloprotease increases the inflammatory cell recruitment and the release of inflammatory cytokines, which aggravates airway inflammation [25]. In addition, MMP-9 induces the degradation of the extracellular matrix, resulting in airway remodeling in asthma [26]. Asthma patients showed significantly elevated MMP-9 expression compared with the healthy controls [27]. Therefore, MMP-9 is considered an important therapeutic target for the control of asthma. Previous studies have demonstrated that MMP-9 expression is modulated by mitogen-activated protein kinases (MAPKs) [28], which are involved in crucial steps in various pathophysiological processes. In the development of asthma, MAPK phosphorylation induces the production of inflammatory cytokines, chemokines, and MMPs [29]. Therefore, suppression of MAPK phosphorylation is implicated as an important step in the inhibition of asthma development. In this study, the phosphorylation of ERK and JNK was significantly elevated in OVA-induced asthmatic mice, which eventually caused increases in cytokine release and MMP-9 expression. In contrast, low-dose irradiation markedly decreased their phosphorylation compared with that in the OVA-induced asthmatic mice, and this decreased was accompanied by a significant reduction in cytokine release and MMP-9 expression. These results indicate that low-dose irradiation suppresses asthmatic responses via inhibition of MMP-9 expression induced by phosphorylation of ERK and JNK.

Overall, low-dose-rate radiation exposure did not induce harmful effects in the development of asthma at dose levels of ≤1 Gy, but attenuated the asthmatic responses induced by the OVA challenge. These results indicate that although low-dose-rate irradiation could prove difficult as a therapeutic strategy for asthma, chronic exposure to low-dose radiation attenuates the asthmatic responses induced by OVA without exacerbating the disease progression. In conclusion, these results may contribute to reducing the concerns of the public about radiation exposure by corroborating the safety of low-dose radiation for the treatment of asthmatic disease.

## Supporting Information

S1 Checklist (PDF)

S1 Table Hematological values. For hematopoietic injury evaluation, 12 mice were randomly assigned to three groups of four mice each and were exposed to sham, 0.3 Gy (0.554 mGy/h), or 1 Gy (1.818 mGy/h) radiation. The low-dose-rate irradiation did not induce any toxicologically significant changes in mortality and clinical signs. Peripheral blood counts were analyzed for myelotoxicity. There were no significant adverse effects on hematology in any irradiated group.
(DOCX)
