# Supplementary material for: Continuous Exposure to Low-Dose-Rate Gamma Irradiation Reduces Airway Inflammation in Ovalbumin-Induced Asthma
Source: PLoS One. 2015 Nov 20;10(11):e0143403. doi: 10.1371/journal.pone.0143403 (PMC4654498; doi:10.1371/journal.pone.0143403)
Supplement: S1 Table — For hematopoietic injury evaluation, 12 mice were randomly assigned to three groups of four mice each and were exposed to sham, 0.3 Gy (0.554 mGy/h), or 1 Gy (1.818 mGy/h) radiation. The low-dose-rate irradiation did not induce any toxicologically significant changes in mortality and clinical signs. Peripheral blood counts were analyzed for myelotoxicity. There were no significant adverse effects on hematology in any irradiated group. (DOCX) [file pone.0143403.s002.docx]

S1 Table. Hematological values

| Indices | Sham | 0.3Gy | 1Gy |
| --- | --- | --- | --- |
| White blood cell (k/μl) | 2.75±0.38 | 2.41±0.23 | 1.80±0.19 |
| Neutrophil (k/μl) | 0.17±0.03 | 0.23±0.04 | 0.18±0.03 |
| Lymphocyte (k/μl) | 2.38±0.29 | 1.94±0.26 | 1.56±0.21 |
| Red blood cell (M/μl) | 10.16±0.29 | 10.26±0.26 | 9.98±0.28 |
| Hemoglobin (g/dl) | 8.95±0.35 | 9.48±0.49 | 8.7±0.18 |
| Platelet (k/μl) | 780.25±23.74 | 730±43.95 | 713.5±49.75 |
